# Proteomic Analysis of Mitochondria-Enriched Fraction Isolated from the Frontal Cortex and Hippocampus of Apolipoprotein E Knockout Mice Treated with Alda-1, an Activator of Mitochondrial Aldehyde Dehydrogenase (ALDH2)

**DOI:** 10.3390/ijms18020435

**Published:** 2017-02-17

**Authors:** Aneta Stachowicz, Rafał Olszanecki, Maciej Suski, Katarzyna Głombik, Agnieszka Basta-Kaim, Dariusz Adamek, Ryszard Korbut

**Affiliations:** 1 Chair of Pharmacology, Jagiellonian University Medical College, 31-531 Krakow, Poland; stachowicz.aneta@gmail.com (A.S.); macieksuski@gmail.com (M.S.); mfkorbut@cyf-kr.edu.pl (R.K.); 2 Department of Experimental Neuroendocrinology, Institute of Pharmacology, Polish Academy of Science, 31-343 Krakow, Poland; glombik@if-pan.krakow.pl (K.G.); basta@if-pan.krakow.pl (A.B.-K.); 3 Chair of Pathomorphology, Jagiellonian University Medical College, 31-531 Krakow, Poland; mnadamek@cyf-kr.edu.pl

**Keywords:** ALDH2, mitochondria, Alzheimer’s disease, apolipoprotein E, brain

## Abstract

The role of different genotypes of apolipoprotein E (apoE) in the etiology of Alzheimer’s disease is widely recognized. It has been shown that altered functioning of apoE may promote 4-hydroxynonenal modification of mitochondrial proteins, which may result in mitochondrial dysfunction, aggravation of oxidative stress, and neurodegeneration. Mitochondrial aldehyde dehydrogenase (ALDH2) is an enzyme considered to perform protective function in mitochondria by the detoxification of the end products of lipid peroxidation, such as 4-hydroxynonenal and other reactive aldehydes. The goal of our study was to apply a differential proteomics approach in concert with molecular and morphological techniques to elucidate the changes in the frontal cortex and hippocampus of apolipoprotein E knockout (apoE^−/−^) mice upon treatment with Alda-1—a small molecular weight activator of ALDH2. Despite the lack of significant morphological changes in the brain of apoE^−/−^ mice as compared to age-matched wild type animals, the proteomic and molecular approach revealed many changes in the expression of genes and proteins, indicating the impairment of energy metabolism, neuroplasticity, and neurogenesis in brains of apoE^−/−^ mice. Importantly, prolonged treatment of apoE^−/−^ mice with Alda-1 led to the beneficial changes in the expression of genes and proteins related to neuroplasticity and mitochondrial function. The pattern of alterations implies mitoprotective action of Alda-1, however, the accurate functional consequences of the revealed changes require further research.

## 1. Introduction

Alzheimer’s disease (AD) is the most widespread neurodegenerative disorder in the elderly, characterized by irreversible loss of cortical neurons associated with the accumulation of β-amyloid deposits and neurofibrillary tangles (NFTs) formed by aggregates of hyperphosphorylated Tau protein [1,2]. It has been recently shown that oxidative stress and multi-faceted dysfunction of mitochondria take part in the pathogenesis of neurodegeneration [3,4]. Indeed, impaired energy metabolism with decreased activity of mitochondrial electron transport chain enzymes (mainly complex I and IV) and citric acid cycle enzymes (pyruvate dehydrogenase complex, α-ketoglutarate dehydrogenase complex) as well as an increased number of mitochondrial DNA mutations, accompanied by augmented mitochondrial reactive oxygen species (ROS) formation have been observed in animal models of neurodegeneration [4] and in AD patients [5,6]. It has been shown that the ROS-dependent lipid peroxidation results in the generation of electrophilic aldehydes such as 4-hydroxy-2-nonenal (4-HNE), malondialdehyde (MDA), and acrolein, which may fervently react with thiols and amino acid residues in proteins. Importantly, it has been observed, that 4-HNE could mediate oxidation of major mitochondrial proteins (e.g., electron transport chain and citric acid enzymes or its cofactors such as lipoic acid) [7]. Moreover, 4-HNE and accumulation of its protein adducts were implicated in mitochondrial dysfunction and have been postulated to play a role in neurodegenerative processes [8,9,10].

Mitochondrial aldehyde dehydrogenase (ALDH2) plays a main role in the degradation of aldehydes to corresponding non-toxic acids [11]. *N*-(1,3-benzodioxol-5-ylmethyl)-2,6-dichloro -benzamide (Alda-1) is a cell-permeable, small molecular weight activator of ALDH2 [12]. It has been observed that Alda-1 administration shows beneficial action in diseases linked to mitochondrial dysfunction (e.g., acute ischemic injury in the heart [13], atherosclerosis and hepatic steatosis [14], diabetes-induced myocardial dysfunction [15], and parkinsonism [16]). Recently, we have demonstrated attenuation of depressive- and anxiety-like behaviors by Alda-1 in rat model of depression [17]. Interestingly, it has also been reported that pharmacological activation of ALDH2 prevents dysfunction of endothelial cells induced by β-amyloid in vitro [18]. However, there is no information whether prolonged ALDH2 activation by Alda-1 may offer any benefits in animal models of neurodegeneration.

Apolipoprotein E (apoE) is a glycoprotein mainly expressed by brain, liver, spleen, lung, adrenal, ovary, kidney, and muscle. In the brain it is predominantly synthetized by astrocytes and microglia, and involved in cholesterol transport as well as in the development and regeneration of the central nervous system (CNS) [19]. The presence of epsilon4 allele of apoE (apoE4) was found to predispose to the development of AD, possibly by impairing proteolytic break-down of β-amyloid [20]. Interestingly, it has also been shown that apoE4 may augment 4-HNE-dependent modification of mitochondrial proteins and cause mitochondrial dysfunction [21,22]. The apoE knockout mice (apoE^−/−^) are primarily used in atherosclerosis studies since they spontaneously develop hypercholesterolemia, dyslipidemia, and arterial lesions [23,24]. They are not considered as a model of full-blown Alzheimer’s disease, however, they have been shown to develop several neurodegenerative changes relevant to the early stages of AD, such as low-grade synaptic and dendrite loss as well as behavioral alterations and deficits in long-term potentiation (LTP) [25,26]. Importantly, despite such delicate morphological alterations, some biochemical disturbances—increased ROS formation and lipid peroxidation—have been clearly demonstrated in brains of apoE^−/−^ mice [27].

Proteomics represents a valuable tool for the study of mitochondrial pathobiology and may contribute to the better understanding of mitochondrial mechanisms of neurodegenerative disorders [28]. However, so far the proteomic research has not been used to evaluate the changes in the brain mitochondria in apoE^−/−^ mice. Thus, the goal of our study was to apply a differential proteomics approach in concert with molecular and morphological techniques to elucidate the changes in the mitochondria-enriched fractions isolated from the frontal cortex and hippocampus of apoE^−/−^ mice as compared to age-matched wild type animals and in apoE^−/−^ mice upon treatment with Alda-1.

## 2. Results

### 2.1. Biochemistry Results

**Figure 1:**
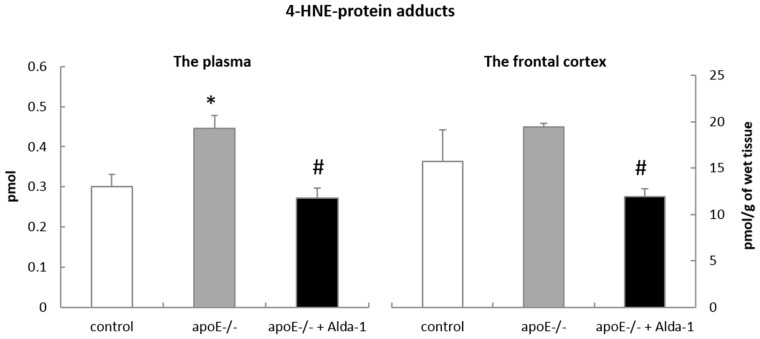
The level of 4-hydroxy-2-nonenal (4-HNE)-protein adducts in the plasma and the frontal cortex of control mice, apolipoprotein E (apoE)^−/−^ mice and Alda-1-treated apoE^−/−^ mice. The data are presented as the mean ± SEM, with *n* = 3 for each group. * *p* < 0.05 vs. control group; # *p* < 0.05 vs. apoE^−/−^ group.

To assess the Alda-1 action in apoE^−/−^ mice, we measured the changes in 4-HNE levels by an enzyme-linked immunosorbent assay (ELISA). Importantly, Alda-1 treatment resulted in a significant decrease of 4-HNE-protein content in the plasma (0.27 vs. 0.44 pmol; F(2,6) = 97.78; *p* < 0.05) and FC (11.91 vs. 19.41 pmol/g; F(2,6) = 10.53; *p* < 0.05) of apoE^−/−^ mice (Figure 1). In addition we observed an increase of 4-HNE-protein content in the plasma (0.44 vs. 0.3 pmol; F(2,6) = 169.81; *p* < 0.05) of apoE^−/−^ mice as compared to control mice (Figure 1).

### 2.2. Histology and Immunohistochemistry Results

**Figure 2:**
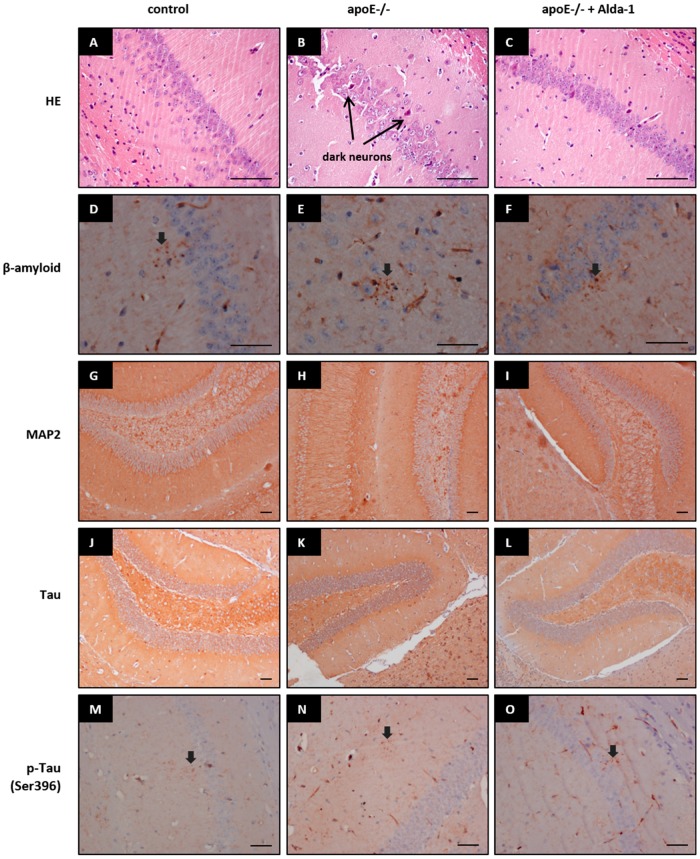
Representative images of hematoxilin and eosin (HE) (**A**–**C**), β-amyloid (**D**–**F**), microtubule-associated protein 2 (MAP2) (**G**–**I**), Tau (**J**–**L**), and p-Tau (Ser396) (**M**–**O**) staining in brains of control mice, apoE^−/−^ mice, and Alda-1-treated apoE^−/−^ mice. Arrows indicate dark neurons, positive stainings of β-amyloid and p-Tau (Ser396), respectively. Scale bars = 500 µm.

Hematoxylin/eosin (HE) staining did not reveal any major changes in the brain structures in control mice, apoE^−/−^ mice, and Alda-1-treated apoE^−/−^ mice. However, apoE^−/−^ mice as compared to wild type animals exhibited signs of the mild tissue injury: some neurons were shrunken and deeply stained (“dark neurons”). These changes were visually slightly less pronounced in brains of Alda-1-treated apoE^−/−^ mice (Figure 2A–C). Immunohistochemistry staining of β-amyloid, microtubule-associated protein 2 (MAP2) (marker of dendrites), and Tau and phospho-Tau (Ser396) (marker of neurofibrillary tangles) did not show any differences between brain structures of wild type mice, apoE^−/−^ mice, and Alda-1-treated apoE^−/−^ mice (Figure 2D–O).

### 2.3. mRNA Expression of Factors Related to Mitochondrial Biogenesis, Apoptosis, Inflammation, and Neuroplasticity

We measured the mRNA expression of selected, important factors related to mitochondrial biogenesis (*PGC1-α*, *Tfam*, *Nrf1*, *CYTB*, *COX1*, *COX3*, *ND1*, *ND4*, *ATP6*), apoptosis (*Bax*, *Bcl-2*, *Bcl2l1*, *Casp8*, *Casp9*, *Casp3*), inflammation (*IL-1β*, *IL-6*, *NF-κB*), neuroplasticity (*Bdnf*, *Nog*, *Bmp4*), and the pathogenesis of Alzheimer disease (*Cdk5*, *Cdk5r1*, *Gsk3a*, *Gsk3b*) in the hippocampus and the frontal cortex of C57BL/6J mice, apoE^−/−^ mice, and Alda-1-treated apoE^−/−^ mice. Both in the frontal cortices (FCx) and the hippocampi (Hp) of apoE^−/−^ mice as compared to controls we observed significant decreases in genes expression related to neurogenesis (*Bdnf*, *Nog*). In addition, apoE^−/−^ mice exhibited decreased *Bcl-2* and *Nrf1* mRNA expression in the Hp and the FCx, respectively (Table 1). Alda-1 administration led to a slight increase in gene expression related to neurogenesis (*Nog*), mitochondrial biogenesis (*CYTB*, *ND1*), and apoptosis (*Bax*, *Gsk3b*) in the Hp of apoE^−/−^ mice (Table 2). In the FCx of apoE^−/−^ mice, Alda-1 treatment resulted in the reduction of *Bmp4* mRNA expression (Table 2).

### 2.4. Mitochondrial Protein Expression Assessed by iTRAQ Method

We investigated mitochondrial protein expression in the FCx and the Hp of control mice, apoE^−/−^ mice, and Alda-1-treated apoE^−/−^ mice using isobaric tag for relative quantitation (iTRAQ method). Collectively, 20 and 34 differentially expressed proteins in the FCx and Hp of apoE^−/−^ mice, respectively, were detected by iTRAQ/mass spectrometry, as compared to control group (Table 3 and Table 4). Alda-1 administration led to 2 and 10 differentially expressed proteins in the FCx and Hp of apoE^−/−^ mice, respectively (Table 5 and Table 6).

## 3. Discussion

Several lines of evidence suggest that altered functioning of apoE may aggravate aldehyde modification of mitochondrial proteins, which may result in mitochondrial dysfunction and neurodegeneration [5,6,21]. In the present work, we focused on the effect of Alda-1 activation of ALDH2—an enzyme responsible for the detoxification of toxic aldehydes—on the protein content of mitochondria-enriched fractions isolated from the frontal cortex and hippocampus of apolipoprotein E knockout mice (apoE^−/−^). Moreover, the proteomic approach was supported by molecular and morphological techniques. This is the first report about the molecular and proteomic effects of pharmacological activation of ALDH2 in CNS structures in apoE^−/−^ mice. The main finding of our study is that Alda-1 administration led to the beneficial changes in the expression of genes and proteins related to neuroplasticity and mitochondrial function.

Although the most recognized function of mitochondrial ALDH2 is the degradation of acetaldehyde in the metabolism of ethanol, it can also efficiently oxidize other reactive aldehydes, especially 4-HNE, to non-toxic acids. Indeed, in our hands, Alda-1 caused a significant decrease in 4-HNE-protein content in the plasma and the frontal cortex of apoE^−/−^ mice, which might depend on ALDH2 activation. However, the exact confirmation of Alda-1 influence on ALDH2 activity regarding the metabolism of 4-HNE to related acids does require further investigations including direct measurements of ALDH2 activity and tissue levels of corresponding acids.

In line with some previous reports [25,26], we observed delicate signs of the nonspecific mild tissue injury in brains of apoE^−/−^ mice. The number of shrunken and deeply stained neurons (“dark neurons”) was higher in brains apoE^−/−^ mice. It should be noted however, that the presence of such changes may depend on the lot of animals, as they were absent in apoE^−/−^ mice studied by Anderson et al. [29]. Moreover, according to some reports, the dark neurons may rather reflect histological artifacts than trustworthy markers of tissue injury [30]. In keeping with the majority of reports [25,26], we did not reveal any changes in tissue contents of β-amyloid, Tau, p-Tau (Ser396), and microtubule-associated protein 2 (MAP2). In our study, we focused on the changes on the levels of the gene/protein expression occurring in the absence of morphologically overt tissue injury.

A growing amount of evidence points to the impairment of neuroplasticity in the pathogenesis of Alzheimer's disease [31,32,33]. Importantly, mitochondria perform a key role in the regulation of neuroplasticity and maintenance of cellular calcium homeostasis [34]. Brain-derived neurotrophic factor (BDNF) is a main regulator of synaptic plasticity, long-term memory, as well as neuronal survival and differentiation [35,36]. Moreover, BDNF is able to streamline mitochondrial respiratory coupling and increase ATP synthesis [37]. Interestingly, it has been observed that lower levels of BDNF in the plasma and the cerebrospinal fluid were presented in the early stages of AD and mild cognitive impairment (MCI) [38]. In keeping with these reports, we showed diminished *Bdnf* mRNA expression in the FCx and Hp of apoE^−/−^ mice, as compared to wild type animals. In turn, noggin (NOG) and bone morphogenetic protein 4 (BMP4) have been recognized as potent regulators of neurogenesis. It is known that BMP4 is related to decreased neurogenesis and increased gliogenesis, while expression of BMP4 inhibitor, NOG, is linked to increased neurogenesis [39] and therefore plays a significant role in the process of learning and memory [40]. Disturbances in hippocampal neurogenesis have been connected to memory deficits and cognitive impairment in the early stages of AD [41]. In this study, *Nog* mRNA expression was decreased both in the FCx and Hp of apoE^−/−^ mice. Importantly, Alda-1 administration led to the increase in *Nog* mRNA expression in the Hp and the decrease in *Bmp4* mRNA expression in the FCx.

Proteomic analysis using iTRAQ and mass spectrometry methods also revealed changes in protein expression related to the impairment of neuroplasticity in the Hp of apoE^−/−^ mice: we observed decreased expression of collapsin response mediator protein 1 (CRMP1), heterogeneous nuclear ribonucleoprotein K (hnRNP K), calcium-dependent secretion activator 1 (CAPS-1), and 2′,3′-cyclic-nucleotide 3′-phosphodiesterase (CNP). CRMP1 is involved in axonal growth and long-term potentiation (LTP) [42], whereas hnRNP K regulates neurite outgrowth, dendritic spine density, and synaptic plasticity in hippocampal neurons [43,44]. In turn, CAPS-1 is a calcium-binding protein engaged in exocytosis of neurotransmitters and neuropeptides, while CNP is required for proper axoglial interactions [45]. Decreased CNP expression, observed in entorhinal and auditory cortex of AD patients, might suggest the impairment in myelination with subsequent synaptic and cognition loss [46]. It is worth highlighting that in our study CNP was a common protein differentially expressed in the Hp of control mice, apoE^−/−^ mice, and apoE^−/−^ mice treated with Alda-1. Importantly, Alda-1 administration up-regulated CNP expression, which may denote its beneficial action in the early stages of AD.

It is well known that a major hallmark of Alzheimer’s disease is the loss of short and long-term memory [13]. Interestingly, we noted down-regulation of calcium/calmodulin-dependent protein kinase type IV (CaMK IV) and basigin (CD147) in the Hp and FCx of apoE^−/−^ mice, respectively. CaMK IV has been demonstrated to participate in the activation of CREB transcription factor, thereby regulating genes responsible for memory and neuronal survival [47]. It can also perform a protective role against neuronal injury [48,49]. In turn, glycoprotein CD147 may regulate the activity of β-amyloid processing enzyme, γ-secretase. It was shown that mice deficient in CD147 showed deficits in memory and spatial learning [50]. It might well be that the decreased expression of the aforementioned proteins could be linked to learning and memory impairment observed in apoE^−/−^ mice in the Morris water maze test [51]. In our case, treatment with Alda-1 resulted in the increase in carbonic anhydrase 2 (CA2) expression in the Hp of apoE^−/−^ mice. Interestingly, CA2 has been postulated to modulate hippocampal CA1 neuronal network activity and its downregulation may result in impaired cognition. Moreover, use of CA2 activators has been shown to improve learning and memory [52]. In this regard, Alda-1 activity toward increase of CA2 might represent promising area of research.

A growing amount of evidence points to the presence of mitochondrial dysfunction manifested by energy metabolism impairment and decreased activity of oxidative phosphorylation (OXPHOS) and Krebs cycle enzymes both in animal models of AD and in patients [5,6,53]. Similarly, mostly in the FCx of apoE^−/−^ mice, we have observed diminished expression of OXPHOS proteins (NADH dehydrogenase flavoprotein 1, cytochrome C oxidase subunit 7A2), proteins supporting OXPHOS (mitochondrial aspartate glutamate carrier 1), and proteins involved in Krebs cycle (succinyl-CoA synthetase subunit α, fumarate hydratase). On the other hand, proteins participating in glycolysis (6-phosphofructokinase type A, hexokinase-1) have been upregulated in the Hp and FCx of apoE^−/−^ mice. However, we have also revealed changes, which may be considered as contradictory: increased expression of ATP synthase subunit δ and cytochrome b-c1 complex subunit 7 in the Hp and FCx of apoE^−/−^ mice, respectively. Clearly, further investigations are required to clarify an interpretation of above effects.

Interestingly, in the FCx of apoE^−/−^ mice we have found decreased mRNA expression of nuclear respiratory factor 1 (*Nrf1*), which is a transcription factor responsible for the activation of expression of many factors involved in energy metabolism, cellular respiration, and replication and transcription of mitochondrial DNA [54]. Diminished expression of peroxisome proliferator-activated receptor gamma coactivator 1-α (*PGC-1α*) and downregulation of *Nrf1* mRNA have been attributed to the impairment of mitochondrial biogenesis in transgenic mouse model of AD [53]. In our case, Alda-1 administration led to the increased expression of proteins that participated in OXPHOS at gene (mitochondrially encoded NADH dehydrogenase 1 (*ND1*), cytochrom b (*CYTB*)), and protein level (NADH dehydrogenase 1α subcomplex subunit 10) in the FCx of apoE^−/−^ mice, which may suggest the stimulation of mitochondrial biogenesis.

Recent reports emphasize the role of glutamate-mediated excitotoxicity in the pathogenesis of neurodegenerative diseases, such as Alzheimer’s disease, Huntington disease, and amyotrophic lateral sclerosis (ALS). Our results indicate that proline-rich transmembrane protein 2 (PRRT2) was down-regulated in the FCx of apoE^−/−^ mice. PRRT2 is a transmembrane protein found in glutamatergic neurons, where it interacts with synaptosomal-associated protein 25 (SNAP25), which inhibits glutamate release and neuronal hyperexcitability. Thus, diminished expression of PRRT2 may lead to decreased interactions with SNAP25, which could potentially result in the increased release of glutamate [55]. Importantly, Alda-1 administration led to the upregulation of excitatory amino acid transporter 2 (EAAT2) both in the Hp and FCx of apoE^−/−^ mice. EAAT2 is crucial for tuning of the glutamate neurotransmission by its rapid removal from the synaptic cleft, thus maintaining the levels of glutamate within safe range [56]. Moreover, it has been shown that reduced EAAT2 function observed in AD is associated with cognitive decline and increased amyloid β production, thus restoring EAAT2 protein function could represent a potential therapeutic approach in AD [57]. It is tempting to speculate that upregulation of EAAT2 by Alda-1 may denote its beneficial action in the early stages of AD.

It is well recognized that oxidative stress plays a crucial role in the etiology and progression of AD [58]. We have observed diminished expression of antioxidant proteins, such as glutathione S-transferase Mu 1 (GST 1-1) and thioredoxin (TRX) in the Hp of apoE^−/−^ mice. GST 1-1 is best known for its ability to detoxify xenobiotics, including 4-HNE, by their conjugation with the reduced form of glutathione (GSH). In turn, TRX acts as an antioxidant by maintaining the reduced form of cellular thiols. Our results are in keeping with previous reports: it has been observed that the lack of TRX2 impairs mitochondrial redox homeostasis and leads to early-onset neurodegeneration [59]; the decreased activity of GST 1-1 has been detected in the Hp of AD patients [60].

Interestingly, some changes observed in apoE^−/−^ mice may represent compensatory mechanisms, for example, Parkinson disease protein 7 homolog (PARK7) was upregulated in the Hp of apoE^−/−^ mice. PARK7 plays multiple cellular roles as a modulator of gene transcription, an antioxidant protein, and a regulator of mitochondrial functions. It can also regulate the activity of complex I in mitochondria and exert a mitoprotective effect [61,62]. Mutations in PARK7 gene are associated with hereditary Parkinson’s disease (PD) [63]. In addition, the increased level of oxidized (inactive) form of PARK7 has been detected in the brain of AD and PD patients [64]. The upregulation of PARK7 in the Hp of apoE^−/−^ mice might be interpreted as a compensatory mechanism, to counteract accelerated oxidative stress and mitochondrial dysfunction presented in the early stages of AD. Similarly, the increase in protein required for synaptic plasticity associated with NMDA receptor signaling-postsynaptic density protein 95 (PSD-95) might represent a compensatory mechanism, supporting synaptic plasticity in the Hp of apoE^−/−^ mice [65].

It should also be noted that not all actions of Alda-1 could be interpreted unambiguously in terms of stimulation of neurogenesis, neuroplasticity, and mitogenesis—for example, in our case, Alda-1 upregulated glycogen synthase kinase 3β (*Gsk3b*) and proapoptotic factor *Bax* mRNA expression in the Hp of apoE^−/−^ mice. It has been shown that increased expression of the active GSK3B form was associated with the formation of senile plaques and neurofibrillary tangles in AD patients [66]. Clearly, further research is needed to clarify the physiological meaning of Alda-1 effects on mRNA and protein of *Gsk3b* and *Bax* in the brain of apoE^−/−^ mice. The main aim of our study was to elucidate the proteomic and molecular changes in the FCx and Hp of apoE^−/−^ mice upon treatment with Alda-1. However, our findings cannot determine whether the changes in gene or protein expression are due to direct or indirect effects of Alda-1 administration in apoE^−/−^ mice. Moreover, the obvious limitation of our study is a lack of corresponding behavioral data, which would significantly increase the impact of the biochemical/molecular and proteomic findings. Clearly, correlation between molecular/proteomic and functional data requires further research.

## 4. Materials and Methods

### 4.1. Animal Experiments

All animal procedures were performed in accordance to the guidelines from Directive 2010/63/EU of the European Parliament on the protection of animals used for scientific purposes and approved by the Jagiellonian University Ethical Committee on Animal Experiments (no. 73/2011, 8 June 2011). Animal experiments were carried out as previously described [14]. Briefly, three groups of animals were studied: control group (C57BL/6J mice, without treatment, on chow diet, *n* = 10), apoE^−/−^ mice from Taconic (Ejby, Denmark) (without treatment, on chow diet, *n* = 10), and apoE^−/−^ mice treated with Alda-1 (Tocris Bioscience, Bristol, UK, *n* = 10) at the age of eight weeks and administered to mice for four months at a dose of 5 mg per kg of body weight per day and mixed without heating with chow diet. At the age of six months mice were euthanized 5 min after injection of fraxiparine (1000 UI; Sanofi-Synthelabo, Paris, France), and the blood from the right ventricle was collected and entire brains or hippocampi (Hp) and frontal cortices (FCx) were dissected on ice-cold glass plates.

### 4.2. Histology and Immunohistochemistry of the Brain

The brain tissue samples were formalin fixed, embedded in paraffin, and 2 µm paraffin sections were stained with the hematoxilin-eosin method or used for immunohistochemistry, as previously described [14]. The following antibodies were used: 1:100 ANTI-MAP2 (sc-20172, Santa Cruz, Dallas, TX, USA), 1:100 ANTI-β-amyloid (A5213, Sigma, Darmstadt, Germany), 1:100 ANTI-Tau (A0024, Dako, Denmark), and 1:100 ANTI-phospho-Tau (Ser396) (SAB4504557, Sigma, Darmstadt, Germany).

### 4.3. Biochemical Methods

The blood was centrifugated at 1000 g-force at 4 °C for 10 min and the plasma was stored in −80 °C until assayed. The FCx were submerged in a PBS buffer (137 mmol/L NaCl, 2.7 mmol/L KCl, 10 mmol/L Na_2_HPO_4_, 1.8 mmol/L KH_2_PO_4_) and homogenized using high-speed shaking (120 s) in plastic tubes with stainless steel beads in TissueLyser II apparatus (Qiagen, Hilden, Germany). The action of Alda-1 was assessed indirectly by the evaluation of the changes in tissue 4-HNE levels measured by an enzyme-linked immunosorbent assay (ELISA) using commercially available kits (MBS027502, MyBioSource, San Diego, CA, USA). The equality of variance and the normality of the data were checked and then one-way analysis of variance (ANOVA) with the Tukey’s post-hoc test implemented in Statistica 10.0 software (StatSoft@Polska, Krakow, Poland) was used for statistical analysis of the data. *p* < 0.05 was considered as statistically significant.

### 4.4. Real Time (RT)-PCR

Real time (RT)-PCR experiments were performed as previously described [14]. Briefly, total RNA was isolated from the homogenized mouse FCx and Hp using RNeasy Fibrous Tissue Mini Kit (Qiagen, Hilden, Germany) according to the manufacturer’s instructions. Relative gene expression analysis in the FCx and Hp was carried out using 7900HT fast real-time PCR System (Applied Biosystems, Foster City, CA, USA) in triplicate with GAPDH, 18S, ACTB, B2M, and GUSB as internal reference genes. The commercially available TaqMan^®^ Gene Expression Assays (Applied Biosystems) were used as follows: Bax (Mm00432051_m1), Bcl-2 (Mm00477631_m1), Bcl2l1 (Mm00437783_m1), Bdnf (Mm04230607_s1), Bmp4 (Mm00432087_m1), Neurog2 (Mm00437603_g1), Nog (Mm01297833_s1), Casp3 (Mm01195085_m1), Casp8 (Mm00802247_m1), Casp9 (Mm00516563_m1), Cdk5 (Mm00432447_g1), Cdk5r1 (Mm00438148_s1), Gsk3a (Mm01719731_g1), Gsk3b (Mm00444911_m1), IL-1β (Mm00434228_m1), IL-6 (Mm00446190_m1), IL-10 (Rn00563409_m1), NFkB (Mm00476361_m1), Ppargc1a (Mm01208835_m1), Pparg (Mm01184322_m1), CYTB (Mm04225271_g1), ND1 (Mm04225274_s1), ND4L (Mm04225294_s1), COX1 (Mm04225243_g1), COX3 (Mm04225261_g1), ATP6 (Gm10925-Mm03649417_g1), Nrf1 (Mm01135606_m1), and Tfam (Mm00447485_m1). Reactions were performed using TaqMan^®^ Gene Expression Master Mix (Applied Biosystems), according to the manufacturer’s instructions. A threshold value (C_t_) for each sample was set in the exponential phase of PCR, and the ΔΔ*C*_t_ method [67] was used for statistical data analysis carried out by Data Assist v3.01 software (Applied Biosystems).

### 4.5. iTRAQ Method

#### 4.5.1. Sample Preparation

The isolation of mitochondria-enriched fraction (mitochondria with synaptosomes) from the freshly-harvested Hp and FCx was performed at 4 °C, as previously described [14]. Mitochondria-enriched pellets were resuspended in 0.5 mL of lysis buffer (7 M urea, 2 M thiourea, 4% 3-(3-Cholamidopropyl)dimethylammonio-1-propanesulfonate hydrate (CHAPS), 1% dithiothreitol (DTT), the mix of protease inhibitors (Sigma)), vortexed, incubated at 25 °C for 30 min and then centrifuged at 12,000× *g*-force for 15 min. The protein concentration was determined in the harvested supernatant using the Bradford method [68]. One hundred micrograms of calculated protein content of each sample was precipitated overnight with ice-cold acetone (Sigma) (1:6 *v*:*v*). Samples were centrifuged at 12,000× *g*-force for 10 min at 4 °C. Acetone was carefully removed and precipitates were air-dried for 10 min. Subsequently, samples were dissolved, reduced, and alkylated, as recommended by iTRAQ protocol (ABSciex, Framingham, MA, USA). Proteins were digested with Trypsin (Promega, Mannheim, Germany) overnight, with 1:50 (*w*:*w*) ratio at 37 °C. Samples were labeled with iTRAQ reagents as recommended by producer and ordered as follows: control: 113, 114, 115 and apoE^−/−^: 116, 117, 118 or apoE^−/−^: 113, 114, 117 and Alda-1-treated: 118, 119, 121. Labeled samples were combined and dried in vacuum concentrator (Eppendorf, Hamburg, Germany). Next, trypsin-digested peptides were dissolved in 5% acetonitrile (ACN), 0.1% trifluoroacetic acid (TFA) and purified with C18 MacroSpin Columns (Harvard Apparatus, Holliston, MA, USA). Eluate was dried in a vacuum concentrator, reconstituted in 5% ACN, 0.1% formic acid (FA), and subjected to strong cation exchange (SCX) fractionation. Samples were loaded onto previously conditioned SCX Macrospin columns (Harvard Apparatus), after which flow-through fraction and 11 consecutive injections of the eluent buffer, which consisted of 5, 10, 20, 40, 60, 80, 100, 150, 200, 300, 500 mM ammonium acetate in 5% ACN, 0.1% FA, respectively, were collected by centrifugation (1 min, 2000 g-force). Thus, the labeled peptides from each sample were distributed across 12 SCX fractions.

#### 4.5.2. LC-MS Analysis

Samples were concentrated on a trap column (Acclaim PepMap100 RP C18 75 µm i.d. ×2 cm column (Thermo Scientific, Waltham, MA, USA). Each fraction was then injected on-line on PepMap100 RP C18 75 µm i.d. ×15 cm column (Thermo Scientific Dionex) and peptides were separated in 90 min 7%–55% B phase linear gradient (A phase − 2% ACN and 0.1% FA; B phase − 80% ACN and 0.1% FA) with a flow rate of 300 nL/min by UltiMate 3000 HPLC system (Thermo Scientific Dionex) and applied on-line to a Velos Pro (Thermo Scientific) dual-pressure ion-trap mass spectrometer. The main working nanoelectrospray ion source (Nanospray Flex, Thermo Scientific) parameters were as follows: ion spray voltage 1.7 kV and capillary temperature 250 °C. Spectra were collected in full scan mode (400–1500 Da, 50 ms AT), followed by five Higher energy Collisional Dissociation (HCD) (20 ms AT), MS/MS scans (each consists of two microscans, 100 ms AT) of the five most intense ions from the preceding survey full scan under dynamic exclusion criteria. Collected data were analyzed by the X!Tandem (The GPM Organization) and Comet search algorithms and validated with PeptideProphet and iProphet under Trans-Proteomic Pipeline (TPP) suite of software (Institute for Systems Biology, Seattle, WA, USA). Search parameters were set as follows: taxonomy: mouse (UniProtKB/Swiss-Prot), enzyme: trypsin, missed cleavage sites allowed: 2, fixed modification: Methylthio(C), variable modifications: Methionine oxidation(M), iTRAQ8plex(K), iTRAQ8plex(N-term), iTRAQ8plex(Y); parent mass error −1.5 to +3.0 Da and peptide fragment mass tolerance: 0.7 Da. Quantitative information of the peptides data was extracted from the HCD MS^2^ data by Libra software under Trans-Proteomic Pipeline (TPP). Peptide False Discovery Rate (FDR) was estimated by Mayu (TPP) and peptide identifications with FDR below 2% were considered as correct matches. DanteR software [69] was used for statistical analysis of iTRAQ-labeled peptides. In brief, replicate peptides were aggregated to unique peptides while the corresponding reporter ion intensities were summed up. The dataset was normalized using linear regression and ANOVA was performed at the peptide level using a robust linear model with minimum 2 and maximum 500 peptides. Finally, the Benjamini and Hochberg False Discovery Rate (FDR) correction was used to adjust *p*-values. The mass spectrometry proteomics data have been deposited to the ProteomeXchange Consortium via the PRIDE [70] partner repository with the dataset identifier PXD004022.

## 5. Conclusions

In conclusion, despite the lack of significant morphological changes, differential proteomic approach revealed many changes in mitochondrial protein expression, indicating the impairment of energy metabolism, neuroplasticity, and neurogenesis in the brains of apoE^−/−^ mice. The treatment of apoE^−/−^ mice with ALDH2 activator, Alda-1, led to the beneficial changes in the expression of genes and proteins related to neuroplasticity and mitochondrial function. The accurate functional consequences of the revealed alterations as well as the detailed mechanism of direct and indirect Alda-1 actions require further research.

## Figures and Tables

**Table 1:** The statistically significant differences in the relative gene expression between apoE^−/−^ group vs. wild type animals in the hippocampus and the frontal cortex (*p* < 0.05, *n* = 3).

Gene Name	Description	Fold Change	*p*-Value
**The Hippocampus**			
*Bcl-2*	B-cell lymphoma 2	−1.30	0.0467
*Bdnf*	Brain-derived neurotrophic factor	−1.27	0.0278
*Bmp4*	Bone morphogenetic protein 4	−1.69	0.0091
*Nog*	Noggin	−1.41	0.0096
**The Frontal Cortex**			
*Bdnf*	Brain-derived neurotrophic factor	−1.29	0.0448
*Nog*	Noggin	−1.39	0.0174
*Nrf1*	Nuclear respiratory factor 1	−1.21	0.0096

**Table 2:** The statistically significant differences in the relative gene expression between Alda-1-treated apoE^−/−^ group vs. untreated apoE^−/−^ group in the hippocampus and the frontal cortex (*p* < 0.05, *n* = 3).

Gene Name	Description	Fold Change	*p*-Value
**The Hippocampus**			
*Bax*	Bcl-2-like protein 4	1.22	0.0303
*CYTB*	Cytochrom b	1.33	0.0258
*Gsk3b*	Glycogen synthase kinase 3β	1.24	0.0058
*ND1*	Mitochondrially encoded NADH dehydrogenase 1	1.35	0.0312
*Nog*	Noggin	1.20	0.0268
**The Frontal Cortex**			
*Bmp4*	Bone morphogenetic protein 4	−1.28	0.0366

**Table 3:** Differentially expressed proteins in the frontal cortex mitochondria of apoE^−/−^ group vs. wild type animals (*p* < 0.05, *n* = 3).

No.	Protein	UniProtKB Accession No.	Unique Peptides	Total Peptides	Fold Change
1	Microtubule-actin cross-linking factor 1	Q9QXZ0	2	2	1.59
2	AP-3 complex subunit δ-1	O54774	2	8	1.30
3	Cytochrome b-c1 complex subunit 7	Q9D855	2	3	1.28
4	Histone H4	P62806	6	28	1.26
5	ATP-dependent 6-phosphofructokinase, muscle type	P47857	6	15	1.24
6	Myelin basic protein	P04370	10	235	1.18
7	Metabotropic glutamate receptor 3	Q9QYS2	4	8	1.18
8	Tubulin β-3 chain	Q9ERD7	12	130	1.11
9	Spectrin α chain, non-erythrocytic 1	P16546	89	416	1.06
10	Tubulin β-4 B chain	P68372	11	356	1.06
11	Calcium-binding mitochondrial carrier protein Aralar1	Q8BH59	15	73	−1.13
12	NADH dehydrogenase (ubiquinone) flavoprotein 1, mitochondrial	Q91YT0	7	18	−1.19
13	Pyruvate carboxylase, mitochondrial	Q05920	2	5	−1.21
14	Solute carrier family 12 member 5	Q91V14	10	46	−1.23
15	Dynein light chain 1, cytoplasmic	P63168	5	17	−1.23
16	Succinyl-CoA ligase (ADP/GDP-forming) subunit α, mitochondrial	Q9WUM5	3	25	−1.26
17	Ubiquitin-60S ribosomal protein L40	P62984	5	57	−1.26
18	Cytochrome c oxidase subunit 7A2, mitochondrial	P48771	2	7	−1.33
19	Basigin	P18572	2	4	−1.41
20	Proline-rich transmembrane protein 2	E9PUL5	2	5	−1.72

**Table 4:** Differentially expressed proteins in the hippocampus mitochondria of apoE^−/−^ group vs. wild type animals (*p* < 0.05, *n* = 3).

No.	Protein	UniProtKB Accession No.	Unique Peptides	Total Peptides	Fold Change
1	Coronin-2B	Q8BH44	2	3	1.73
2	Protein deglycase DJ-1	Q99LX0	5	20	1.32
3	Synaptoporin	Q8BGN8	4	8	1.23
4	ATP synthase subunit δ, mitochondrial	Q9D3D9	3	18	1.23
5	Spectrin β chain, erythrocytic	P15508	3	9	1.23
6	MICOS complex subunit Mic25	Q91VN4	2	3	1.21
7	Ubiquitin-60S ribosomal protein L40	P62984	4	51	1.21
8	Tubulin α-1 chain	A2AQ07	7	33	1.17
9	Tropomyosin α-3 chain	P21107	3	16	1.17
10	Paralemmin-1	Q9Z0P4	7	33	1.16
11	Disks large homolog 4	Q62108	5	19	1.14
12	Pyruvate dehydrogenase E1 component subunit β, mitochondrial	Q9D051	16	86	1.14
13	Creatine kinase B-type	Q04447	19	306	1.11
14	Calcium/calmodulin-dependent protein kinase type II subunit α	P11798	25	329	1.10
15	Tubulin β-4 B chain	P68372	11	249	1.09
16	Heat shock cognate 71 kDa protein	P63017	37	351	1.08
17	Hexokinase-1	P17710	33	246	1.07
18	Spectrin β chain, non-erythrocytic 1	Q62261	72	351	1.07
19	ADP/ATP translocase 1	P48962	22	217	−1.11
20	Guanine nucleotide-binding protein G(I)/G(S)/G(T) subunit β-1	P62874	12	134	−1.11
21	Calcium-dependent secretion activator 1	Q80TJ1	16	56	−1.14
22	Fumarate hydratase, mitochondrial	P97807	10	38	−1.15
23	Glutathione S-transferase Mu 1	P10649	15	76	−1.15
24	Dihydropyrimidinase-related protein 1	P97427	8	32	−1.15
25	Thioredoxin	P10639	3	11	−1.18
26	Ras-related protein Rab-1A	P62821	6	46	−1.20
27	Actin-related protein 2	P61161	6	13	−1.24
28	Mitochondrial 2-oxoglutarate/malate carrier protein	Q9CR62	6	16	−1.26
29	Heterogeneous nuclear ribonucleoprotein K	P61979	3	13	−1.28
30	α-endosulfine	P60840	2	3	−1.30
31	Calcium/calmodulin-dependent protein kinase type IV	P08414	2	4	−1.34
32	Guanine nucleotide-binding protein G(i) subunit α-2	P08752	4	13	−1.37
33	Myelin basic protein	P04370	11	138	−1.43
34	2′,3′-cyclic-nucleotide 3′-phosphodiesterase	P16330	32	210	−1.47

**Table 5:** Differentially expressed proteins in the frontal cortex mitochondria of Alda-1-treated apoE^−/−^ group vs. untreated apoE^−/−^ group (*p* < 0.05, *n* = 3).

No.	Protein	UniProtKB Accession No.	Unique Peptides	Total Peptides	Fold Change
1	NADH dehydrogenase ubiquinone 1α subcomplex subunit 10, mitochondrial	Q99LC3	5	10	1.27
2	Excitatory amino acid transporter 2	P43006	12	111	1.12

**Table 6:** Differentially expressed proteins in the hippocampus mitochondria of Alda-1-treated apoE^−/−^ group vs. untreated apoE^−/−^ group (*p* < 0.05, *n* = 3).

No.	Protein	UniProtKB Accession No.	Unique Peptides	Total Peptides	Fold Change
1	Myelin basic protein	P04370	11	123	1.37
2	Carbonic anhydrase 2	P00920	4	24	1.27
3	Myelin proteolipid protein	P60202	4	48	1.25
4	2′,3′-cyclic-nucleotide 3′-phosphodiesterase	P16330	22	141	1.25
5	Sodium/potassium-transporting ATPase subunit α-2	Q6PIE5	22	180	1.11
6	Excitatory amino acid transporter 2	P43006	15	183	1.09
7	Spectrin β chain, non-erythrocytic 1	Q62261	64	303	−1.06
8	Spectrin α chain, non-erythrocytic 1	P16546	98	606	−1.06
9	Actin, aortic smooth muscle	P62737	24	182	−1.09
10	OCIA domain-containing protein 2	Q9D8W7	2	7	−1.40

## Abbreviations

4-HNE	4-hydroxy-2-nonenal
ACN	acetonitrile
AD	Alzheimer’s disease
ALDH2	mitochondrial aldehyde dehydrogenase
apoE	apolipoprotein E
BDNF	brain-derived neurotrophic factor
BMP4	bone morphogenetic protein 4
CA2	carbonic anhydrase 2
CaMK IV	calcium/calmodulin-dependent protein kinase type IV
CAPS-1	calcium-dependent secretion activator 1
CD147	basigin
CHAPS	3-(3-Cholamidopropyl)dimethylammonio-1-propanesulfonate hydrate
CNP	2′,3′-cyclic-nucleotide 3′-phosphodiesterase
CRMP1	collapsin response mediator protein 1
DTT	dithiothreitol
EAAT2	excitatory amino acid transporter 2
FA	formic acid
FCx	frontal cortices
GSK3B	glycogen synthase kinase 3β
GST 1-1	glutathione S-transferase Mu 1
hnRNP K	heterogeneous nuclear ribonucleoprotein K
Hp	hippocampi
LTP	long-term potentiation
NOG	noggin
NRF1	nuclear respiratory factor 1
OXPHOS	oxidative phosphorylation
PARK7	Parkinson disease protein 7 homolog
PD	Parkinson’s disease
SCX	strong cation exchange
TFA	trifluoroacetic acid
TRX	thioredoxin
